# Serum microRNA profiling in patients with glioblastoma: a survival analysis

**DOI:** 10.1186/s12943-017-0628-5

**Published:** 2017-03-11

**Authors:** Hua Zhao, Jie Shen, Tiffany R. Hodges, Renduo Song, Gregory N. Fuller, Amy B. Heimberger

**Affiliations:** 1 Department of Epidemiology, The University of Texas MD Anderson Cancer Center, 1155 Pressler Street, Houston, TX 77030 USA; 2 Department of Neuro-Surgery, The University of Texas MD Anderson Cancer Center, Houston, TX 77030 USA; 3 Department of Pathology, The University of Texas MD Anderson Cancer Center, Houston, TX 77030 USA

**Keywords:** Serum microRNAs, Glioblastoma, and prognosis

## Abstract

Because circulating microRNAs (miRNAs) have drawn a great deal of attention as promising novel cancer diagnostics and prognostic biomarkers, we sought to identify serum miRNAs significantly associated with outcome in glioblastoma patients. To do this, we performed global miRNA profiling in serum samples from 106 primary glioblastoma patients. The study subjects were randomly divided into two sets: set one (*n* = 40) and set two (*n* = 66). Using a Cox regression model, 3 serum miRNAs (miR-106a-5p, miR-182, and miR-145-5p) and 5 serum miRNAs (miR-222-3p, miR-182, miR-20a-5p, miR-106a-5p, and miR-145-5p) were identified significantly associated with 2-year patient overall survival and disease-free survival (*P* < 0.05) in both sets and the combined set. We then created the miRNA risk scores to assess the total impact of the significant serum miRNAs on survival. The high risk scores were associated with poor patient survival (overall survival: *HR* = 1.92, 95% CI: 1.19, 10.23, and disease-free survival: *HR* = 2.03, 95%CI: 1.24, 4.28), and were independent of other clinicopathological factors. Our results suggest that serum miRNAs could serve as prognostic predictors of glioblastoma.

## Background

microRNAs (miRNAs) are small endogenous mediators of RNA interference and key regulatory components of many biological processes required for an organism’s development, cell specialization, and homeostasis [1, 2]. Many miRNAs exhibit tissue-specific patterns of expression and are deregulated in various cancers, where they can be either oncogenic (oncomirs) or tumor suppressive. In glioblastoma, the most common malignant brain tumor in adults [3–5], miRNA dysregulation plays diverse roles in various aspects of gliomagenesis, ranging from cancer stem cell regulation (miRs-7, 9, 10a/10b, 17–92, 124/137, 125a/125b, 182-5p, and 326), cell cycle control (miRs-15b, 34a, and 221/222), apoptosis (miRs-21, 26a, 101, 146b-5p, 153, 181a/181b, 196a/196b, 218, 381, 451), and angiogenesis (miRs-93 and 296), to immune modulation (miR-124, 138, 142-3p) [6–12].

miRNAs from tumors can be secreted within membrane vesicles (exosomes) [13, 14] or directly into the blood [15, 16], indicating that miRNAs probably play a key role in intracellular communication. As such, miRNAs are increasingly being profiled as biomarkers for diseases, including cancers [17–23], circulating in the bloodstream. Profiling of miRNAs circulating in serum or plasma may be particularly useful in glioblastoma patients, given the risks of sequential surgery and biopsy, and the lack of readily usable biomarkers to predict clinical outcome. Previous attempts have been made to assess circulating miRNAs in relation to glioblastoma prognosis [24–30]. However, due to small sample size and a heterogeneous patient population, prognostic impact could not be elucidated. In the current study, we perform miRNA profiling in serum samples from 106 primary glioblastoma patients from The University of Texas M. D. Anderson Cancer Center and identify a panel of miRNAs associated with patient outcome.

## Findings

### Patient population

Study subjects were recruited from The University of Texas M. D. Anderson Cancer Center (Houston, TX) beginning in April 2013 under an Institutional Review Board approved protocol. Written informed consent was obtained from each study subject. Demographic and clinical data for the study subjects were obtained from medical record review. Serum samples were selected retrospectively at the time of analysis according to the following requirements: 1) the patients were newly diagnosed with glioblastoma defined by WHO 2007 criteria [31]; 2) serum was available in sufficient volume for RNA isolation; and 3) demographic, clinical, and follow-up data for the patients were available. All patients received initial standard of care treatment of resection, radiation, and chemotherapy. Case patients with recurrent glioblastoma or prior cancer (except for non-melanoma skin cancer) were excluded from the study. A total of 106 glioblastoma patients were included in the study.

Serum miRNAs were isolated using the miRNeasy kit (Qiagen), with minor modifications. miRNA expression profiling was carried out using the nCounter Human v2 miRNA Expression Assay (NanoString Technologies). For each patient, disease-free survival was calculated from the date of disease diagnosis to the date of disease recurrence/progression, or to date of death from disease, or to the last follow-up date. Overall survival was calculated from the date of disease diagnosis to the date of death from disease or to the date of last patient follow-up visit. The associations between 2-year survival and serum miRNA expression levels were estimated as hazard ratios (HRs) and 95% confidence intervals (CIs) for each miRNA using the Cox proportional hazards model, adjusted for age, sex, race, smoking status, Karnofsky Performance Scale (KPS) score, timing of blood draw, *IDH1* mutation status, and body mass index (BMI). Patients with survival times over 24 months were censored at 24 months in the Cox regression analysis. The proportional hazards assumption for identified significant miRNAs was tested within each set using the Schoenfeld residuals. miRNAs with evidence for non-proportionality were deleted. The combined 3-miRNA risk score (for overall survival) and 5-miRNA risk score (for disease-free survival) for each patient was derived by linear combination of the product of the reference-normalized expression level of each miRNA times its Cox regression corresponding coefficient. All patients were dichotomized by the median risk score, and individuals with a risk score higher or lower than the median were classified as high- or low-risk groups, respectively. In all statistical analyses, a *P* value < 0.05 was considered significant. Then, an estimate of the false discovery rate (*q* value) was calculated to take into account the multiple comparisons for any miRNA significantly associated with 2-year overall survival or disease-free survival in all two sets. A low q value (*q* < 0.15) is an indication of high confidence in a result. miRNAs that were significantly in both two sets were deemed as the significant miRNAs.

### Serum miRNAs predicting overall survival

Basic characteristics of the patient cohort at baseline are shown in Table 1. A total of 106 patients with primary glioblastoma were included in the study. We divided the study subjects into two sets: set one and set two. The set one included 40 glioblastoma patients, 20 who had survived at least 24 months and 20 who died within 24 months after diagnosis. The set two included 66 glioblastoma patients, 53 who had survived at least 24 months and 13 who died within 24 months after diagnosis. Overall, basic characteristics were very similar between two sets. Overall, the mean age was 58 years, and 66% of the study subjects were male. Most of them were Caucasians (94.3%), and nearly half of them had a history of cigarette smoking. Two-thirds of the study subjects had a KPS score of at least 90. Of 67 study subjects for whom *IDH1* mutation data was available, 9 (13.4%) carried an *IDH1* mutation. In terms of the timing of obtaining blood samples, nearly half of the study subjects (46.2%) had their blood drawn immediately after surgery; in 24.5%, blood was drawn after or during adjuvant chemotherapy; and in 26.4%, blood was drawn after or during chemoradiation. The median follow-up interval was 19 months. During the follow-up period, 68 study subjects (64.8%) experienced disease recurrence or progression.

**Table 1:** Basic characteristics of the patient cohort with primary glioblastoma

Variables	Set one (*N* = 40)	Set two (*N* = 66)	Combined set (*N* = 106)
Vital status			
Alive	20 (50%)	53 (84.1%)	73 (68.9%)
Dead	20 (50%)	13 (19.7%)	33 (31.1%)
Sex			
Female	15 (37.5%)	25 (37.9%)	40 (37.7%)
Male	25(62.5%)	41 (62.1%)	66 (62.3%)
Ethnicity			
White	37 (92.5%)	63 (95.4%)	100 (94.3%)
Non-White	3 (7.5%)	3 (4.54%)	6 (5.7%)
Smoking history			
Ever a smoker	18 (45.0%)	30 (45.5%)	48 (45.3%)
Never a smoker	17 9(42.5%)	29 (43.9%)	46 (43.4%)
Unknown	5 (12.5%)	5 (7.6%)	10 (9.4%)
Recurrence/progression			
Yes	26 (65.0%)	42 (63.6%)	68 (64.8%)
No	14 (35.0%)	23 (36.4%)	37 (35.2%)
KPS score			
100	8 (20.0%)	15 (22.7%)	23 (21.7%)
90	20 (50.0%)	27 (40.9%)	47 (44.3%)
80	5 (12.5%)	14 (21.2%)	19 (17.9%)
< 80	7 (17.5%)	10 (15.2%)	17 (16.0%)
Timing of blood draw			
Newly diagnosed, post surgery	18 (45.0%)	31 (47.0%)	49 (46.2%)
Newly diagnosed, immediately post or during radiation therapy	0 (0.0%)	3 (4.5%)	3 (2.8%)
Newly diagnosed, immediately post or during adjuvant chemotherapy	12 (30.0%)	14 (21.2%)	26 (24.5%)
Newly diagnosed, immediately post or during chemoradiation	10 (25.0%)	18 (27.3%)	28 (26.4%)
*IDH1* mutation status			
Negative	24 (60.0%)	34 (51.5%)	58 (54.7%)
Positive	3 (7.5%)	6 (9.1%)	9 (8.5%)
unknown	13 (32.5%)	26 (39.4%)	39 (36.8%)
Age, mean (range)	59 (45, 69)	56 (46, 70)	58 (45, 70)
BMI, mean (range)	28.5 (22.0, 35.4)	27.4 (21.5, 36.1)	28.1 (21.5, 36.1)
Follow-up time, median (range)	18 (2, 23)	19 (3, 24)	19 (2, 24)

Abbreviations: *KPS* Karnofsky Performance Scale, *BMI* body mass index

Of 812 human miRNAs we were capable of detecting, 187 miRNAs were expressed in at least 80% of the patients’ serum samples. After imputation with minimum observed values for each serum miRNA, we first attempted to identify serum miRNAs associated with 2-year overall survival times (Table 2). In set one, 5 serum miRNAs (miR-630, 106a-5p, 182, 15b-5p, and 145-5p) were associated with 2-year overall survival. Among them, 2 serum miRNAs (miR-630, 106a-5p) were significantly associated with a lower probability of 2-year overall survival, and 3 serum miRNAs were significantly associated with a higher probability of 2-year overall survival (miR-182, 15b-5p and 145-5p). In set two, 5 serum miRNAs (miR-106a-5p, 20a-5p, 17-5p, 182, and 145-5p) were associated with 2-year overall survival. Among them, 3 serum miRNAs (miR-106a-5p, 20a-5p, 17-5p) were significantly associated with a lower probability of 2-year overall survival, and 2 serum miRNAs (miR-182, 145-5p) were significantly associated with a higher probability of 2-year overall survival. Three serum miRNAs were identified as significantly associated (*P* <0.05) with overall survival in both sets; these were miR-106a-5p, miR-182, and miR-145-5p. After adjusting for multiple comparisons, the associations were remained significant (*q* < 0.15). These associations with 2-year overall survival were further confirmed in a meta-analysis by combining the samples from two sets. Increased levels of miR-106a-5p and decreased levels of miR-182 and miR-145-5p were significantly associated with a lower probability of 2-year overall survival. Additionally, in the meta-analysis, we observed 2 more serum miRNAs, miR-20a-5p and miR-17-5p, whose increased levels were significantly associated with an increased hazard of death (*P* = 0.040 and 0.043, respectively).

**Table 2:** Serum miRNA expression associated with 2-year overall survival in patients with primary glioblastoma

	Set one (*N* = 40)		Set two (*N* = 66)		Combined (*N* = 106)	
microRNAs	Adj. HR (95% CI)^a^	*P* value	Adj. HR (95% CI)^a^	P value	Adj. HR (95% CI)^a^	*P* value
miR-630	1.69 (1.10, 4.57)	0.035	1.54 (0.74, 3.48)	0.473	1.62 (0.89, 3.41)	0.258
**miR-106a-5p**	**1.68 (1.04, 4.83)**	**0.043**	**1.61 (1.02, 3.94)**	**0.046**	**1.71 (1.07, 3.63)**	**0.038**
miR-20a-5p	1.60 (0.95, 4.95)	0.090	1.72 (1.05, 3.69)	0.045	1.69 (1.06, 3.79)	0.040
miR-17-5p	1.57 (0.94, 5.01)	0.096	1.81 (1.05, 3.98)	0.046	1.70 (1.05, 4.01)	0.043
**miR-182**	**0.66 (0.28, 0.98)**	**0.049**	**0.67 (0.26, 0.98)**	**0.048**	**0.60 (0.29, 0.92)**	**0.037**
miR-15b-5p	0.62 (0.30, 0.98)	0.049	0.86 (0.42, 1.65)	0.458	0.76 (0.40, 1.52)	0.402
**miR-145-5p**	**0.57 (0.23, 0.92)**	**0.036**	**0.54 (0.25, 0.90)**	**0.037**	**0.59 (0.29, 0.88)**	**0.028**

^a^Adjusted by age, sex, ethnicity, smoking status, KPS score, timing of blood drawn, *IDH1* mutation status, and BMI

Data in bold are significant miRNAs

Abbreviations: *HR* hazard ratio, *CI* confidence interval

### Serum miRNAs predicting disease-free survival

Next we investigated the associations between serum miRNAs and 2-year disease-free survival (Table 3). In set one, 7 serum miRNAs (miR-922, 222-3p, 20a-5p, 106.5p, 182, 548a, 145-5p) were associated with 2-year disease-free survival. Among them, 4 serum miRNAs (miR-922, 222-3p, 20a-5p, 106a-5p) were significantly associated with a lower probability of 2-year disease-free survival, and 3 serum miRNAs (miR0182, 548a, 145-5p) were significantly associated with a higher probability of 2-year disease-free survival. In set two, 7 serum miRNAs (miR222-3p, 20a-5p, 106a-5p, 124a, 182, 371-5p, 145-5p) were associated with 2-year disease-free survival. Among them, 3 serum miRNAs (miR-222-3p, 20a-5p, 106a-5p) were significantly associated with a lower probability of 2-year disease-free survival, and 4 serum miRNAs (miR-124a, 182, 371a-5p, 145-5p) were significantly associated with a higher probability of 2-year disease-free survival. Five serum miRNAs (*P* <0.05) showed a significant association with 2-year disease-free survival in both sets: miR-222-3p, miR-182, miR-20a-5p, miR-106a-5p, and miR-145-5p. After adjusting for multiple comparisons, the associations were remained significant (*q* < 0.15). Their significant associations with 2-year disease-free survival were further confirmed in a meta-analysis by combining the samples from two sets. Increased levels of miR-222-3p, miR-20a-5p, and miR-106a-5p, and decreased levels of miR-182 and miR-145-5p were significantly associated with a lower probability of 2-year disease-free survival.

**Table 3:** Serum miRNA expression associated with 2-year disease free survival in patients with primary glioblastoma

	Set one (*N* = 40)		Set two (*N* = 66)		Combined (*N* = 106)	
microRNAs	Adj. HR (95% CI)^a^	*P* value	Adj. HR (95% CI)^a^	*P* value	Adj. HR (95% CI)^a^	*P* value
miR-922	1.78 (1.09, 4.62)	0.040	1.39 (0.65, 3.72)	0.506	1.61 (0.75, 3.29)	0.369
**miR-222-3p**	**1.77 (1.07, 4.85)**	**0.043**	**1.61 (1.02, 3.94)**	**0.046**	**1.71 (1.07, 3.63)**	**0.038**
**miR-20a-5p**	**1.66 (1.06, 4.45)**	**0.044**	**1.68 (1.02, 3.72)**	**0.045**	**1.68 (1.05, 3.81)**	**0.040**
**miR-106a-5p**	**1.61 (1.05, 4.39)**	**0.046**	**1.71 (1.09, 3.69)**	**0.039**	**1.72 (1.04, 3.76)**	**0.047**
miR-124a	0.69 (0.27, 1.05)	0.084	0.63 (0.22, 0.97)	0.048	0.65 (0.26, 1.03)	0.062
**miR-182**	**0.68 (0.28, 0.96)**	**0.047**	**0.67 (0.28, 0.97)**	**0.048**	**0.69 (0.30, 0.94)**	**0.043**
miR-548a	0.68 (0.28, 0.99)	0.049	0.70 (0.29, 1.16)	0.242	0.61 (0.32, 1.04)	0.106
miR-371a-5p	0.64 (0.27, 1.26)	0.379	0.62 (0.29, 0.99)	0.049	0.62 (0.30, 1.05)	0.098
**miR-145-5p**	**0.62 (0.28, 0.99)**	**0.049**	**0.56 (0.27, 0.97)**	**0.047**	**0.60 (0.32, 0.92)**	**0.039**

^a^Adjusted by age, sex, ethnicity, smoking status, KPS score, timing of blood drawn, *IDH1* mutation status, and BMI

Data in bold are significant miRNAs

Abbreviations: *HR* hazard ratio, *CI* confidence interval

### miRNA risk scores predicting survival outcomes

**Fig. 1:**
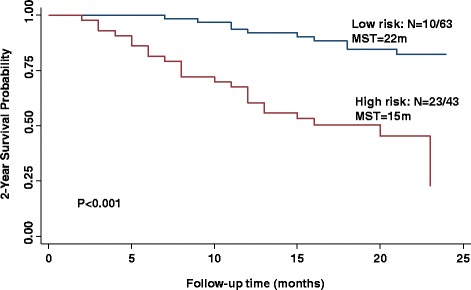
Kaplan-Meier 2-year overall survival estimate for patients with primary glioblastoma grouped by low (*blue line*) and high (*red line*) risk score. *N* = number of patients with an event (death) at 2 years/total number of patients in the dataset. MST = median survival time

**Fig. 2:**
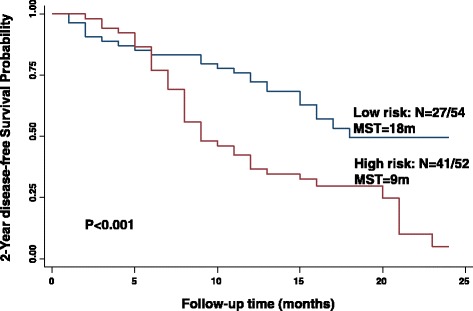
Kaplan-Meier 2-year disease-free survival curves for patients with primary glioblastoma grouped by low (*blue line*) and high (*red line*) risk score. *N* = number of patients with an event (death) at 2 years/total number of patients in the dataset. MST = median survival time

Last, we assessed the combined effects of these serum miRNAs on survival. We generated 2 risk scores, one for overall survival and the other for disease-free survival, using the linear combination of expression levels of these significant miRNAs for each individual based on the median levels of each single miRNA (Table 4). The included miRNAs were miR-106a-5p, 182 and 145-5p for overall survival, and miR-222-3p, 20a-5p, 106a-5p, 182 and 145-5p for disease-free survival. For 2-year overall survival, study subjects with high-risk scores exhibited a significantly increased hazard of death relative to those with low-risk scores (*HR* = 1.92, 95% CI, 1.19, 10.23). The Kaplan-Meier 2-year overall survival curve showed that patients with the high-risk scores had a median survival time (MST) of 15 months, compared with 22 months in the low-risk group (*P* < 0.001) (Fig. 1). For 2-year disease-free survival, study subjects with high-risk scores exhibited a significantly decreased chance for disease-free survival relative to those with low-risk scores (*HR* = 2.03, 95% CI, 1.24–4.28). The Kaplan-Meier 2-year disease-free survival curve showed that patients with the high-risk scores had a median survival time (MST) of 9.0 months, compared with 18 months in the low-risk group (*P* < 0.001) (Fig. 2).

**Table 4:** Serum miRNA risk score associated with 2-year survival in patients with primary glioblastoma

	Risk score	Event, n (%)	Event-free, n (%)	Adj. HRs (95%CI)^*^	*P* value
Overall	Low	10 (30.3%)	53 (72.6%)	reference	
	High	23 (69.7%)	20 (27.4%)	1.92 (1.19, 10.23)	0.008
Disease-free	Low	27 (39.7%)	27 (73.0%)	reference	
	High	41 (60.3%)	10 (27.0%)	2.03 (1.24, 4.28)	2.35E-04

^*^Adjusted by age, sex, ethnicity, smoking status, KPS score, timing of blood drawn, *IDH1* mutation status, and BMI

Abbreviations: *HR* hazard ratio, *CI* confidence interval

Although the idea of using circulating biomarkers, either alone or as an adjunct to genetic and molecular profiling of the tumor, to monitor tumor progression/recurrence and clinical outcome prediction has already entered clinical practice [32, 33], little progress has been made in this context for glioblastoma patients. In the current study, we performed global miRNA profiling in serum from 106 patients with primary glioblastoma and identified a panel of serum miRNAs associated with glioblastoma prognosis.

In recent years, endogenous circulating miRNAs have attracted extensive research interest because they remain stable and are well protected even under harsh conditions. A number of studies have investigated the potential role of circulating miRNAs in glioma survival and clinical outcomes [24, 25, 27, 29, 30]. For example, Zhi et al. reported that up regulation of serum miR-20a-5p, miR-106a-5p, and miR-181b-5p was associated with advanced clinical stages of astrocytoma, and high expression of serum miR-19a-3p, miR-106a-5p, and miR-181b-5p was significantly associated with poor patient survival among astrocytoma patients [29]. In our study, we found that elevated levels of serum miR-106a-5p and miR-20a-5p were associated with poor survival. Thus, our results support their findings. Both miR-20a and miR-106a have previously been linked to gliomagenesis. Overexpression of miR-20a and miR-106a was observed in glioma stem cells, and such overexpression could promote glioma invasiveness by directly targeting tissue inhibitor of metalloproteinases-2 (TIMP-2) [34]. Knockdown of either miR-20a or miR-106a was observed to increase TIMP-2 expression and consequently inhibit glioma stem cell invasion. Xiao et al. found that high levels of plasma miR-182 were associated with poor overall survival (*P* = 0.034) and disease-free survival (*P* = 0.013) among glioma patients [24]. However, in our study, we found that decreased levels of miR-182 were associated with poor overall survival (*P* = 0.037) and disease-free survival (*P* = 0.043) among glioblastoma patients. The reasons for this discrepancy are unclear, but it may be due differences in the study populations. Xiao’s study included patients with a mixture of glioma types ranging from WHO grade I to IV, whereas our study was confined to glioblastoma patients. Using oncogenomic analyses of 470 miRNAs profiled by The Cancer Genome Atlas (TCGA) program, Kouri et al. revealed that miR-182 was the only miRNA that was associated with favorable patient prognosis and temozolomide (TMZ) sensitivity, and was associated with a less aggressive oligoneural subclass of glioblastoma [35]. In another study from the same group, miR-182 was found to play a key role in apoptosis, growth, and differentiation in glioblastoma [36], and overexpression of miR-182 in orthotopic glioblastoma xenografts led to reduced tumor burden and increased animal survival.

In addition to the miRNAs mentioned above, we observed that elevated serum levels of miR-222-3p and miR-17-5p, and decreased levels of miR-145-5p, were associated with a poor glioblastoma prognosis. Although similar associations have not been reported in the literature yet, these miRNAs have been linked to various phenotypes of glioblastoma. For example, overexpression of miR-222 leads to increased DNA damage, tumor growth promotion, and a poor glioblastoma prognosis [37]. On the other hand, suppression of miR-222 expression inhibits glioma cell proliferation and invasion. Elevated levels of miR-17 were found in glioblastoma samples and were negatively related to patients’ survival [38]. The level of miR-17 is also increased in glioblastoma spheroids, which are enriched in tumor-initiating cells (TICs) or tumor stem-like cells (TSCs) [39]. In contrast, miR-145 is an important repressor of pluripotency in embryonic stem cells and acts as a tumor suppressor in a variety of cancers, including glioma [40]. Lee et al. found that the low expression of miR-145 in glioblastoma was correlated with poor patient prognosis [41]. Thus, our findings on serum miR-222-3p, miR-17-5p, and miR-145-5p are consistent with their biological functions in glioblastoma carcinogenesis.

## Conclusions

In summary, we identified a panel of serum miRNAs associated with 2-year overall survival and disease-free survival among patients with glioblastoma. Our study provides evidence to support the role of circulating miRNAs in predicting survival with glioblastoma. Further validation from larger independent studies and further characterization of these candidate serum miRNAs are warranted.
